# Menstrual function among women exposed to polybrominated biphenyls: A follow-up prevalence study

**DOI:** 10.1186/1476-069X-4-15

**Published:** 2005-08-09

**Authors:** Stephanie I Davis, Heidi Michels Blanck, Vicki S Hertzberg, Paige E Tolbert, Carol Rubin, Lorraine L Cameron, Alden K Henderson, Michele Marcus

**Affiliations:** 1 Department of Epidemiology, Rollins School of Public Health, Emory University, 1518 Clifton Road, Alanta, GA, 30322, USA; 2 Graduate Division of Biological and Biomedical Sciences, Emory University, 1462 Clifton Road, NE, Atlanta, Georgia, 30322, USA; 3 Department of Biostatistics, Rollins School of Public Health, Emory University, 1518 Clifton Road, NE, Atlanta, Georgia, 30322, USA; 4 Department of Environmental and Occupational Health, Rollins School of Public Health, Emory University, 1518 Clifton Road, NE, Atlanta, Georgia, 30322, USA; 5 Division of Environmental Hazards and Health Effects, National Center for Environmental Health, Centers for Disease Control and Prevention, 4770 Buford Highway, MS F-46, Atlanta, Georgia, 30341, USA; 6 Division of Environmental and Occupational Epidemiology, Michigan Department of Community Health, P.O. Box 30195, Lansing, Michigan, 48909, USA

## Abstract

**Background:**

Alteration in menstrual cycle function is suggested among rhesus monkeys and humans exposed to polybrominated biphenyls (PBBs) and structurally similar polychlorinated biphenyls (PCBs). The feedback system for menstrual cycle function potentially allows multiple pathways for disruption directly through the hypothalamic-pituitary-ovarian axis and indirectly through alternative neuroendocrine axes.

**Methods:**

The Michigan Female Health Study was conducted during 1997–1998 among women in a cohort exposed to PBBs in 1973. This study included 337 women with self-reported menstrual cycles of 20–35 days (age range: 24–56 years). Current PBB levels were estimated by exponential decay modeling of serum PBB levels collected from 1976–1987 during enrollment in the Michigan PBB cohort. Linear regression models for menstrual cycle length and the logarithm of bleed length used estimated current PBB exposure or enrollment PBB exposure categorized in tertiles, and for the upper decile. All models were adjusted for serum PCB levels, age, body mass index, history of at least 10% weight loss in the past year, physical activity, smoking, education, and household income.

**Results:**

Higher levels of physical activity were associated with shorter bleed length, and increasing age was associated with shorter cycle length. Although no overall association was found between PBB exposure and menstrual cycle characteristics, a significant interaction between PBB exposures with past year weight loss was found. Longer bleed length and shorter cycle length were associated with higher PBB exposure among women with past year weight loss.

**Conclusion:**

This study suggests that PBB exposure may impact ovarian function as indicated by menstrual cycle length and bleed length. However, these associations were found among the small number of women with recent weight loss suggesting either a chance finding or that mobilization of PBBs from lipid stores may be important. These results should be replicated with larger numbers of women exposed to similar lipophilic compounds.

## Background

In 1973, the fire retardant chemical, FireMaster^®^, was mistaken for NutriMaster^®^, a magnesium oxide-based cattle feed supplement, and was inadvertently introduced into cattle feed (Michigan Chemical Corporation, St. Louis, MI). Thousands of farm families and farm product consumers were exposed to this commercial mixture of polybrominated biphenyl (PBB) congeners throughout Michigan. Concern about possible adverse health effects led to the establishment of the Michigan PBB Long-Term Study in 1976 by the Michigan Department of Community Health (MDCH). The incident is described in detail elsewhere [1,2].

From 1997–1998, the Michigan Female Health Study (MFHS) was conducted to assess whether PBB exposure disrupted endocrine function among cohort women. Only one rhesus monkey study was found, suggesting that PBBs may be associated with longer menstrual cycles [3]. Animal studies of structurally similar polychlorinated biphenyls (PCBs) have found that menstrual cycle length was either not different [4-7] or longer [8] with Aroclor 1248 [4,8] or Aroclor 1254 [5-7] dosing. Bleed length was marginally longer in the higher Aroclor 1254 dose groups [5-7]. Human PCB studies are also inconsistent. Shorter cycle length was associated with indices of PCB-contaminated fish consumption [9]. When summed over measures of selected PCB congeners, cycle length was longer with higher total PCB levels in a large multicenter cohort [10], but not among Southeast Asian immigrants [11]. Cooper et al reported that bleed length was not associated with PCBs [10], but Yucheng women, with cooking oil exposure to PCBs, reported abnormal bleeding more often than controls [12].

Endocrine regulation is a complex process and may be disrupted at many points. The immune and neuroendocrine systems are integrated in a network of the ovarian, thyroid, thymus, and adrenal axes. This network is regulated or disrupted through feedforward and feedback loops of the hypothalamus, the pituitary, and associated end-organs of the neuroendocrine axes [13-15].

Cycle length and bleed length, although nonspecific, are markers for the reproductive status of women undergoing cyclical ovulation and endometrial angiogenesis under hormonal feedback and control [16-20]. This study assessed whether serum PBB levels were associated with these markers of menstrual cycle function among women still menstruating in the year before interview.

## Methods

### The Michigan Female Health Study design and data collection

The release of PBBs into the Michigan food chain was limited in geographic distribution and in duration due to the environmental remediation of quarantined farms and the food chain. Michigan PBB cohort enrollment data, along with blood samples for exposure biomonitoring that included PBB and PCB, were collected from 1976–1987 by the MDCH.

The study design of the MFHS is a follow-up prevalence study [21]. It is a hybrid study of an exposure cohort followed prospectively to assess the prevalence of current menstrual cycle outcomes. Data were collected for the MFHS during August 1997 through April 1998 by computer-assisted telephone interview. Participants were asked about reproductive history, menstrual cycle characteristics, physical activity, smoking habits, medication, physician-diagnosed medical conditions, and other health and demographic information. The MFHS was approved by the institutional review boards of Emory University, MDCH, and the Centers for Disease Control and Prevention.

### Eligibility for menstrual cycle analyses

A total of 1020 women in the MFHS had serum levels of PBB available from cohort enrollment. This study was restricted to women who were born before the exposure incident, which is estimated to have occurred as early as July 1, 1973 [22]. In this manner, we restricted the analyses to women solely exposed by food ingestion and not in utero.

**Figure 1:**
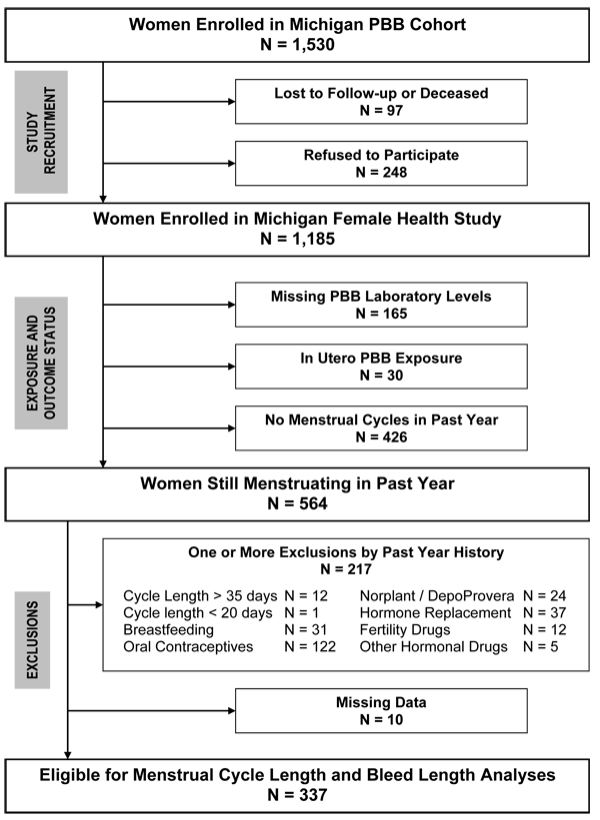
Eligibility for menstrual cycle analysis, Michigan Female Health Study, 1997–1998.

Women were asked to report their usual non-pregnancy related cycle length during the past year, defined as the number of days from the first day of menstrual bleed to the first day of the next menstrual bleed. The distribution of menstrual cycle length across the reproductive lifespan represents a mixture between normal ovulatory cycles and short or long anovulatory bleeding episodes at both age extremes [23-25]. Long cycles in the right tail are influenced by older women in perimenopausal status [24,25] with more frequent ovulatory failure [23]. Among women aged 25–44 years, Metcalf observed that 93.6% of all cycles were between 20–35 days in length with a pattern of consistently ovulatory cycles [23]. We lacked laboratory confirmation and referred to the women in this study as having normal, not ovulatory, cycles. We decomposed the distribution of cycle length into an approximately symmetric part centered about 28 days (n = 338) and a long right tail (n = 12). We restricted our analysis to women with cycle lengths ranging from 20–35 days (n = 337), excluding a subject with a 14-day cycle length (Figure 1). Other studies define normal cycles from 21–35 days [26], in general agreement with our definition, while others recommend cycle lengths of 18–40 days [25].

We excluded women from menstrual cycle analyses if they indicated any of the following during the past year: no menstrual cycles, menstrual cycles shorter than 20 days or longer than 35 days, breastfeeding, or use of hormone medications. We also excluded ten women with missing data (Figure 1).

We restricted bleed length analyses to the same women who were eligible for cycle length analyses. Women reported their usual length of menstrual bleeding in the past year as a single estimate or a range in days. Bleed length for women reporting a range was taken as the midpoint between the two values.

### Measurement of enrollment serum PBB and PCB concentrations

PCBs and PBBs have 209 theoretically possible congeners. By weight, FireMaster^® ^was 54%–68% PBB 153, a non-coplanar congener. Several coplanar congeners were present in the mixture in smaller amounts [22,27]. Serum concentrations of PBB 153, in parts per billion (ppb), were quantified by gas chromatography with electron capture detection using established protocols. The limit of detection (LOD) was 1 ppb. These PBB 153 measures are indicators of total PBB exposure [28,29].

Up to 1982, total PCBs were quantified as Aroclor 1254 (Montsano Company, St. Louis, MO) levels with the LOD at 5 ppb. Between 1982–1993, Aroclor 1260 (Montsano Company, St. Louis, MO) levels were assessed with the LOD at 3 ppb. We combined initial total PCB levels from the two assay methods, assuming any total below 5 ppb was below the LOD. Only three women (0.9%) had initial Aroclor 1260 levels, all below 3 ppb. Changing PCB analytical methods did not result in misclassification of exposure rank in this sample.

### Estimation of current serum PBB concentration

We hypothesized that current PBB levels were associated with current menstrual function. We used serum PBB enrollment measurements to estimate individual current PBB levels, using an exponential decay model described previously [30]. The original decay model included women who were at least 16 years old at enrollment with initial PBB levels of 2 ppb or higher. They also had at least two nonpregnancy serum samples drawn. The authors found that overall, median half-life was 13.5 years, but decay was slower among women with high enrollment BMI. Parity, age at enrollment, smoking history, and breastfeeding duration were not significant predictors of decay [30]. The estimated levels from the decay model were highly correlated with measured levels (r = 0.92) [31].

We used the same equation specified in the decay model for our estimates. We assigned enrollment PBB levels less than or equal to 1 ppb a value of 0.5 ppb, a value half way between zero and the LOD. This enabled log-transformations [32], and has been shown to be an appropriate imputation technique [33]. We included data from the MDCH archives: linear and quadratic terms for the logarithm of enrollment PBB level, categories of body mass index (BMI) at enrollment (less than/greater than or equal to 23.0 kg/m^2^), and age at enrollment. We also included data from the MFHS: number of full-term pregnancies between enrollment and interview, breastfeeding duration (months), and smoking status (ever/never). Because these data were modeled, we did not impose an LOD on the estimated levels.

### Classification of estimated current PBB exposure

PBB and PCB exposures were ranked in low, middle, and high exposure categories. Tertiles were our primary classification for estimated current PBBs: with low, from zero to 0.06 ppb (n = 112) as referent; middle, from greater than 0.06–0.32 ppb (n = 113); and high, at greater than 0.32 ppb (n = 112). We also performed analyses with estimated current PBBs categorized for highest exposure, retaining the lowest tertile as the referent and classifying the upper decile at greater than 3.08 ppb (n = 35).

### Classification of PBB exposure at cohort enrollment

We categorized laboratory measures of PBB at enrollment into approximate tertiles: at or below the LOD of 1 ppb (n = 111) as referent; from greater than 1 to 3 ppb (n = 120); and greater than 3 ppb (n = 106). We classified enrollment PBB levels greater than 12 ppb (n = 37) as the upper decile, retaining the same referent group.

### Classification of PCB exposure

We used initial serum PCB measurements. We did not attempt to model the decay and bioaccumulation of PCBs due to changes in the laboratory assessment from Aroclor 1254 to Aroclor 1260. We also did not have information on important factors of PCB bioaccumulation, such as fish consumption. We divided PCB exposure into four categories. The lowest referent category included measurements at or below the LOD or 5 ppb (n = 178); the middle category, from greater than 5 to 7 ppb (n = 53); the high category, at greater than 7 ppb (n = 62); and missing (n = 44).

### Statistical analyses

**Figure 2:**
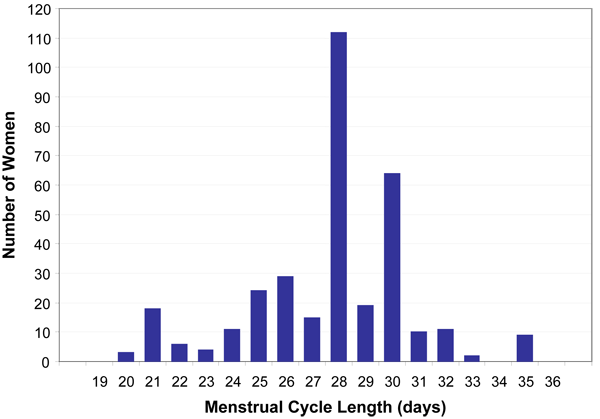
Distribution of menstrual cycle length, Michigan Female Health Study, 1997–1998

**Figure 3:**
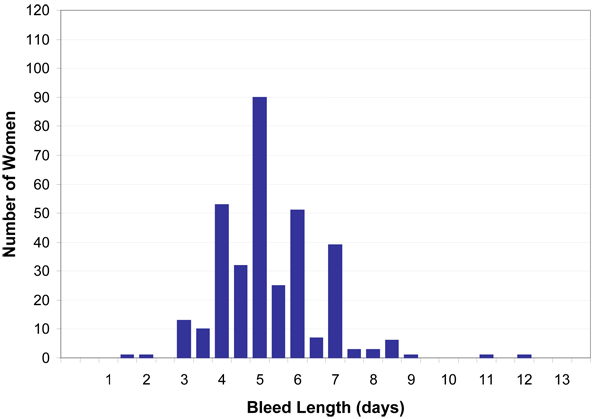
Distribution of bleed length, Michigan Female Health Study, 1997–1998

We performed multiple linear regressions with cycle length and with the natural logarithm of bleed length as continuous outcomes. We examined the distributions of the continuous variables to better meet the assumptions of normality in linear regression models. We did not apply data transformations for age and BMI at interview, or for cycle length restricted to 20–35 days (median 28.0; mean 27.7 days; standard deviation (SD) 2.9; skewness -0.42; kurtosis 0.70) (Figure 2). Bleed length was right-skewed (median 5.0; mean 5.3; SD 1.3; skewness 0.87; kurtosis 2.5) (Figure 3). We log-transformed bleed length to center the distribution (median 1.61; mean 1.63; SD 0.25; skewness -0.37; kurtosis 1.91). We verified that the quantile-quantile (QQ) plot for log-transformed bleed length linearized the curved point pattern we observed in the QQ plot for bleed length. We performed collinearity diagnostics, and centered age and BMI about their respective means (37.8 years and 26.6 kg/m^2^) to reduce inflated variances in the models.

We retained the same covariates in all regression models across both cycle length and bleed length analyses. Categorical covariates included PCBs, usual weekly physical activity, smoking status, educational attainment, income level, and loss of 10% of body weight or more in the past year unrelated to pregnancy. We defined never exercising as low, one or two times per week as moderate, and exercising three or more times per week as high levels of recreational physical activity.

We evaluated interactions of PBB categories with age and BMI at interview, weight loss, and physical activity for all models. Interaction assessment for age or BMI with PBBs included testing linear and quadratic terms. When significant interactions between age and PBB categories were detected, diagnostic tests were repeated with age quintiles. The age range for the first quintile was 24–31 years (n = 59); the second, 32–35 years (n = 70); the third, 36–38 years (n = 64) as the referent group; the fourth, 39–43 years (n = 74); and the fifth, 44–56 years (n = 70). We also evaluated possible additive or synergistic effects between exposures to PBBs and PCBs or cigarette smoking by interaction assessment. We exponentiated point estimates and confidence intervals for the difference in the logarithm of bleed length and reported the converted differences in bleed length as a ratio on the original scale, in days.

The decay model we used to estimate current PBB levels was originally developed using different criteria than the inclusion criteria for this study. We performed sensitivity analyses by repeating our models excluding females less than 16 years at enrollment (n = 140).

We performed secondary analyses for the full distribution of menstrual cycle length, including women in the long right tail. Logistic regression models would not converge due to small cell sizes with long cycles classified as greater than 35 days (n = 12; upper 3%). We redefined long cycles as greater than 30 days (n = 43; upper 10%) and examined the relationship between current estimated PBB exposure and the prevalence of long cycles.

We retained women with self-reported thyroid conditions (n = 39, 11.6%) in analyses to avoid adjusting for possible PBB-related thyroid-mediated menstrual cycle effects [34-41]. We did not include in utero diethylstilbestrol exposure as a covariate or exclusion due to the high number of women reporting unknown exposure status (n = 101, 30.0%). Five women (1.5%) reported having in utero diethylstilbestrol exposure. We performed sensitivity analysis removing these five women from the final models. Most women lacked historical pesticide information (n = 298; 88.4 percent); therefore, it was not included in this analysis.

We performed Pearson partial correlation analyses between cycle length and the logarithm of bleed length, assessing overall associations and correlations stratified by history of weight loss. We also performed correlation analyses between the logarithm of both PBB and PCB, and age in years. All analyses were performed using SAS Version 9.1 (SAS Institute Inc., Cary, NC).

## Results

### Descriptive statistics of the study population

After restricting our sample to women with cycle lengths between 20–35 days, average cycle and bleed lengths were 27.7 days and 5.3 days, respectively. The women, all white, ranged in age from 24 to 56 years. Most never smoked and reported some form of weekly exercise. Educational attainment was evenly distributed across PBB tertiles. Forty-four women (13.0%) reported losing 10% or more of their body weight in the past year (Table 1).

**Table 1:** Characteristics of 337 women,* Michigan Female Health Study, 1997–1998.

Population Characteristics		Distribution
		
Continuous Variables		Point Estimate (Range)
Median PBB level (ppb) measured at cohort enrollment		2.0 (0.5–1490)
Median PBB level (ppb) estimated at study interview		0.1 (0–1005)
Median initial PCB level (ppb) measured in cohort follow-up		5.0 (1.5–78) ^†^
Mean age (years) at study interview		37.8 (24–56)
Mean BMI (kg/m^2^) at study interview		26.6 (16.9–53.1)
		
Categorical Variables		N (%)

Past Year History of Weight Loss = 10%	No	293(86.9%)
	Yes	44 (13.0%)
Amount of weekly exercise	Low (Never)	48 (14.2%)
	Moderate (1 or 2 times)	137 (40.6%)
	High (3 or more times)	152 (45.1%)
Lifetime smoking status	Never Smoker	222 (65.9%)
	Ever Smoker	115 (34.1%)
Educational attainment	High School Grad or Less	132 (39.2%)
	Some College or Technical	110 (32.6%)
	College or Postgrad	95 (28.2%)
Annual Household Income	Less Than $35,000	113 (33.5%)
	At Least $35,000	207 (61.4%)
	Unknown	17 (5.0%)

* Cycle lengths ranging from 20–35 days. ^† ^44 missing PCB levels.

In our sample, 318 women (94.4%) had enrolled in the cohort by the end of 1977. The remaining 19 (5.6%) enrolled over the next 9 years. Using the exponential decay model [30], we extrapolated enrollment levels of PBB over an average of 20.5 years (median: 20.6; 25^th ^%: 20.3; 75^th ^%: 20.9; range: 10.3–22.4; in years) to estimate PBB levels at the time of the MFHS interview. Most women categorized as having low, middle, or high PBB exposure at enrollment were classified the same way by estimated current PBB tertiles (75.7%, 57.5%, and 79.2%, respectively in Table 2). If laboratory LODs were applied to the decay estimates, 277 women (82.2%) would currently have PBB levels below 1 ppb (median: 0.13; mean: 7.73; 25^th ^%: 0.03; 75^th ^%: 0.51; range: 0–1005; in ppb). A total of 86 women were common to our study (25.5% of 337) and the decay study (22.6% of 380).

**Table 2:** Cross tabulation of PBB tertiles from two timeframes, Michigan Female Health Study, 1997–1998.

No. in Tertiles of PBB Exposure Measured at Cohort Enrollment, (Range, ppb)	No. in Tertiles of PBB Exposure Estimated at Time of Study Interview (Range, ppb)			Total No.
		
	Low (0.00–0.06)	Middle (>0.06–0.32)	High (>0.32)	
Low (≤ 1.0)	84	27	0	111
Middle (1.0–3.0)	23	69	28	120
High (>3.0)	5	17	84	106
Total No.	112	113	112	337

We observed digit preferences in reporting menstrual cycle length for 30 days and 7-day multiples, noting peaks at 21 (n = 18), 28 (n = 112), and 30 (n = 64) days (Figure 2). The distribution of bleed length did not exhibit digit preferences. Because we allowed women to report their usual bleed length either directly or in a range, 84 women (24.9%) had half-day estimates (Figure 3).

### Crude associations between PBB, PCB, and population characteristics

Average cycle length did not differ among women when stratified by PBB exposure at enrollment or by PBB exposure estimated at the time of the interview. There was a suggested increase in bleed length with increasing PBB tertiles at enrollment and time of interview (Table 3).

**Table 3:** Menstrual cycle outcomes by PBB tertiles from two time frames, Michigan Female Health Study, 1997–1998.

Outcomes	Tertiles of PBB Estimated at Study Interview (No.)			P * (df)	Tertiles of PBB Measured at Cohort Enrollment (No.)			P * (df)
				
	Low (112)	Middle(113)	High (112)		Low (111)	Middle (120)	High (106)	
Mean Cycle Length (SD), days	27.4 (3.1)	28.0 (2.9)	27.7 (2.8)	0.33 (2)	27.5 (3.0)	27.6 (2.8)	28.0 (3.0)	0.34 (2)
Mean Bleed Length (SD), days^†^	5.0 (1.3)	5.1 (1.3)	5.2 (1.3)	0.36 (2)	4.9 (1.3)	5.1 (1.3)	5.3 (1.3)	0.14 (2)

* ANOVA F test for continuous variables. ^† ^Exponentiated from log(bleed length).

Age and BMI were positively correlated (r = 0.13, p = 0.02). The data suggested that women in the lowest tertile of enrollment PBBs were older and had higher BMIs; conversely, women in the highest tertile of estimated current PBBs were older and had higher BMIs (Table 4). Previously, Blanck et al also noted slower PBB decay among women with higher BMIs [30]. We found little difference in the frequency of weight loss in the past year and in the usual levels of weekly physical activity by PBB tertiles (Table 4).

**Table 4:** Menstrual cycle covariates by PBB tertiles from two time frames, Michigan Female Health Study, 1997–1998.

Covariates	Tertiles of PBB Estimated at Study Interview (No.)			P * (df)	Tertiles of PBB Measured at Cohort Enrollment (No.)			P * (df)
				
	Low (112)	Middle (113)	High (112)		Low (111)	Middle (120)	High (106)	
Continuous Variables								
Mean Age (SD), years	37.7 (6.9)	36.8 (6.1)	38.8 (6.9)	0.07 (2)	38.9 (6.4)	37.1 (6.4)	37.3 (7.2)	0.07 (2)
Mean BMI (SD), kg/m^2^	25.8 (5.4)	26.9 (6.5)	27.0 (6.8)	0.28 (2)	27.5 (6.4)	26.3 (6.4)	25.8 (5.9)	0.12 (2)
Amount of Weekly Exercise								
Low No. (%)	14 (12.5)	17 (15.0)	17 (15.2)	0.27 (4)	13 (11.7)	15 (12.5)	20 (18.9)	0.27 (4)
Moderate No. (%)	38 (33.9)	48 (42.5)	51 (45.5)		41 (36.9)	50 (41.7)	46 (43.4)	
High No. (%)	60 (53.6)	48 (42.5)	44 (39.3)		57 (51.4)	55 (45.8)	40 (37.7)	
Past Year History of Weight Loss = 10%								
Without No. (%)	95 (84.8)	97 (85.8)	101 (90.2)	0.45 (2)	97 (87.4)	103 (85.8)	93 (87.7)	0.90 (2)
With No. (%)	17 (15.2)	16 (14.2)	11 (9.8)		14 (12.6)	17 (14.2)	13 (12.3)	

* ANOVA F test for continuous variables; Chi square test for categorical variables.

The frequency of diagnosed thyroid conditions did not differ across either PBB tertiles estimated at interview or measured at enrollment (p = 0.76 and 0.46, respectively; 2 df). Mean menstrual cycle length and bleed length did not differ when stratified by history of thyroid disorders (p = 0.09 and 0.58, respectively; 1 df). Enrollment PBBs and enrollment PCBs were positively correlated among the 293 women with both measures available (r = 0.12, p = 0.04); however, estimated current PBBs and enrollment PCBs, were not (r = 0.06, p = 0.27). Age was positively correlated with PCBs (r = 0.18, p = 0.002), but not PBBs (enrollment: r = -0.09, p = 0.10; adjusted current: r = 0.02, p = 0.66).

### Multiple regression models for cycle length and bleed length

No grossly influential observations were noted in model diagnostics. The adjusted R^2 ^values for linear regression models were 5% for cycle length and 7% for the logarithm of bleed length.

We observed an association between physical activity and bleed length, but not cycle length, regardless of PBB categories used in the models. Women with high levels of physical activity had bleed lengths 0.92 times shorter than those with moderate levels (95% confidence limits: 0.87, 0.97).

We found no overall association between current estimated PBBs and either menstrual cycle length or bleed length; however, the associations between PBB exposure and menstrual cycle length or bleed length differed for women based on their history of weight loss in the past year. The interaction terms for past year weight loss and PBB tertiles were not statistically significant for cycle length (Table 5). When we considered women with weight loss in the highest decile of estimated current PBB exposure, the interaction term was significant. This small group of women (n = 4) had cycle lengths 3.55 days shorter (95% confidence limits: -6.45, -0.65) than the referent group. We observed a similar association using upper decile enrollment PBB levels with even fewer women with weight loss (n = 3). Their mean cycle lengths were 5.54 days shorter (95% confidence limits: -8.83, -2.26) than their respective referent group. These three were among the four women with weight loss in the upper decile of estimated current PBBs.

**Table 5:** Cycle length by weight loss and PBB tertiles, Michigan Female Health Study, 1997–1998. Adjusted for PCB, BMI and age at interview, physical activity, smoking history, education, income.

	Difference in Cycle Length, (days)					
	
	Without Weight Loss			With Weight Loss		
	
	No.	Diff	(95% CI)	No.	Diff	(95% CI)
Tertiles of PBB Estimated at Study Interview ^§^						
Low	95	0.00	Ref	17	-0.28	(-1.79 to 1.22)
Middle	97	0.35	(-0.48 to 1.18)	16	0.21	(-1.34 to 1.76)
High	101	0.30	(-0.53 to 1.13)	11	-1.04	(-2.86 to 0.77)
Test for Interaction: p = 0.57, 2 df						
Tertiles of PBB Measured at Cohort Enrollment						
Low	97	0.00	Ref	14	0.35	(-1.27 to 1.98)
Middle	103	0.01	(-0.80 to 0.82)	17	-1.02	(-2.53 to 0.48)
High	93	0.54	(-0.30 to 1.39)	13	-0.22	(-1.88 to 1.46)
Test for Interaction: p = 0.45, 2 df						

* Cycle lengths ranging from 20–35 days.

Women with weight loss also exhibited a monotonic increase in bleed length with increasing PBB exposure relative to referent women in the low PBB tertile without weight loss. Among women with weight loss in estimated current PBB tertiles, those in the low tertile had shorter bleed lengths than the referent group with a ratio of 0.82; those in the middle tertile had a bleed length ratio of 0.92; and those in the high tertile had longer bleed lengths with a ratio of 1.27 (test for interaction: p < 0.0001) (Table 6). We observed consistent results in the model examining the upper decile (test for interaction: p = 0.002, data not shown). We also noted PBB-weight loss interactions with enrollment PBB tertiles that were consistent with the models for estimated current PBB tertiles and bleed length (test for interaction: p = 0.08) (Table 6).

**Table 6:** Bleed length by weight loss and PBB tertiles, Michigan Female Health Study, 1997–1998. Adjusted for PCB, BMI and age at interview, physical activity, smoking history, education, income.

	Ratio of Bleed Length *					
	
	Without Weight Loss			With Weight Loss		
	
	No.	Ratio	(95% CI)	No.	Ratio	(95% CI)
Tertiles of PBB Estimated at Study Interview ^§^						
Low	95	1.00	Ref	17	0.82	(0.72 to 0.93)
Middle	97	0.92	(0.85 to 1.01)	16	0.92	(0.80 to 1.06)
High	101	1.00	(0.91 to 1.09)	11	1.27	(1.08 to 1.50)
Test for Interaction: p < 0.0001, 2 df						
Tertiles of PBB Measured at Cohort Enrollment						
Low	97	1.00	Ref	14	0.89	(0.78 to 1.03)
Middle	103	1.03	(0.96 to 1.10)	17	0.98	(0.86 to 1.12)
High	93	1.03	(0.96 to 1.11)	13	1.16	(1.00 to 1.34)
Test for Interaction: p = 0.08, 2 df						

* Exponentiated from difference in log(bleed length).

**Figure 4:**
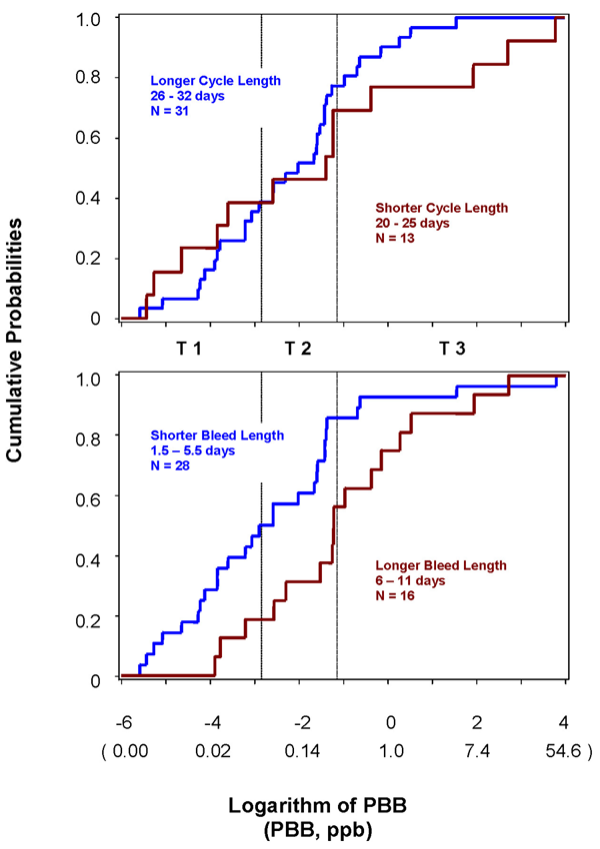
**Cumulative distribution functions for estimated current log(PBB) among women with weight loss**. Shorter cycle length defined as lower 25^th^%. Longer bleed length defined as upper 75^th^%. T1 = Low Tertile; T2 = Middle Tertile; T3 = High Tertile.

We also examined the cumulative distribution functions (CDF) for log-transformed current estimated PBBs. There was no difference in the distribution of PBBs between women who did and did not report past year weight loss (exact two-sided Wilcoxon p = 0.61). We further examined the CDFs for PBB distributions among the 44 women with past year weight loss by menstrual cycle length and bleed length categories (Figure 4). We dichotomized PBB strata for shorter cycle length in the lower quartile (≤ 25 days) and also for longer bleed length in the upper quartile (≥ 6 days). CDFs for PBBs did not differ for women with shorter cycle lengths (Wilcoxon p = 0.63); however, the CDFs for PBBs stratified for longer bleed length were different (Wilcoxon p = 0.006). We observed that women who had longer bleed lengths with past year weight loss were more likely to have higher PBB levels (Figure 4).

Aging one year shortened cycle length by 0.10 day (95 percent confidence interval: -0.14 to -0.05) regardless of the PBB categories included in multiple regression models. Bleed length had a J-shaped relationship with age that was modified by estimated current PBB categories (tertile and decile models, tests for interaction: p = 0.04). Due to the curvilinear effect with age modeled as a continuous variable, a clear dose-response in these models was difficult to discern. Therefore, we repeated tests for these interactions with age quintiles cross-classified with estimated current PBB categories (tertile model: p = 0.03; decile model: p = 0.01). The women who were 24–31 years old in the upper tertile of estimated PBB exposure (n = 17) had bleed lengths 0.79 times shorter than the 36 to 38 year olds in the lowest tertile (n = 20) (95 percent confidence interval: 0.67, 0.93). The 24–31 year olds in the upper decile of exposure (n = 8) had bleed lengths 0.70 times shorter (95 percent confidence interval: 0.57, 0.87). We did not observe the same effect for bleed length with enrollment level PBBs. The MFHS women in the youngest age quintile (n = 59) were 6–13 years old at the time of the Michigan incident.

In our sensitivity analysis of women who were the same age as those in the exponential decay model, we excluded 140 women less than 16 years at enrollment (41.5%). We found that, despite this restriction to 197 women, all significant PBB-weight loss interactions persisted for both cycle length (adjusted PBB for upper decile, p = 0.04; initial PBB for upper decile, p= 0.02) and bleed length (adjusted PBB tertiles and upper decile, p < 0.0001; initial PBB tertile, p = 0.01; initial PBB for upper decile, 0.04). For the PBB-age interaction for bleed length with estimated current PBBs, we no longer noted a significant age interaction (tertile model: p = 0.38; decile model: p = 0.32). This is reasonable due to the age restriction imposed on this even smaller sample of women. In addition, among the 197 women, age was no longer an independent predictor of cycle length.

We tested the interaction between PBBs and PCBs for the full sample of 337 women including 44 women missing PCBs, and for the subset of 293 women with both PBB and PCB measures available. No significant interactions were detected for either enrollment or estimated current PBBs with enrollment PCBs, for either cycle length or bleed length. In our secondary analysis of long cycle length, we found no overall association between PBB exposure and menstrual cycle length greater than 30 days (n = 43; upper 10%).

In addition, the sensitivity analyses removing the five women with in utero DES exposure did not affect our results.

## Discussion

Normal menstrual cyclicity is a marker of successful follicular recruitment and ovulation. Factors such as aging, weight loss, body composition, and physical activity are thought to affect menstrual cycle function through the hypothalamic-pituitary-adrenal axis [42-52]. Physical activity and age were associated with menstrual cycle function in this sample of women. We observed bleed length shortening with increased physical activity that was consistent with the literature [45,46]. We did not observe menstrual cycle lengthening with physical activity as was reported in another sample of Michigan women [47]. Our observation of cycle length shortening with increasing age is supported by the literature as well. Older women who are still menstruating exhibit accelerated development of dominant follicles and shorter mean cycle lengths due to shorter follicular phases [53], and follicle density has been clinically measured and mathematically modeled to decrease with age [54-56].

Weight loss, lactation, and pregnancy mobilize lipophilic chemicals like PBBs, normally resistant to elimination, from adipose tissue into systemic circulation, and potentially induce menstrual cycle and other endocrine changes [57-61]. We found no overall association between PBB exposure and menstrual cycle length or bleed length, but significant interactions between PBB exposure and past year weight loss not related to pregnancy were found for both cycle length and bleed length. Our results were consistent for both estimated current and measured enrollment PBB concentrations. Among women with weight loss, those in the highest tertile of PBB exposure experienced longer bleed length than women in the lowest tertile. Among the women in the lowest PBB tertile with weight loss, we observed shorter bleed length, as might be expected among women with weight loss in the general population [48-51]. We did not observe any differences in cycle length with PBB exposure or weight loss when PBB was categorized in tertiles. However, among the small number of women in the upper decile of PBB exposure who experienced weight loss, cycle length was shorter by 3.5 days. The direction of this effect is consistent with observations of women exposed to PCBs through fish consumption [9], but not with the longer cycles observed by Cooper et al in their multicenter cohort [10]. On the other hand, we observed no association between PCB exposure and menstrual cycle length or bleed length. Multiple testing and small numbers were a major concern. It is possible that our results were due to chance or artifactual findings. We did, however, observe the same cycle length and bleed length differences among women with weight loss when we restricted our analysis to an even smaller subsample of 197 women at least 16 years old at cohort enrollment.

We also observed significant age-related interactions with PBB exposure for bleed length. We found no evidence in the literature that bleed length is a correlate of age among regularly cycling women in the general population. We observed age-related bleed length differences only in association with current estimated PBB exposure, not with measured enrollment PBB levels. The observed age interaction with PBB exposure could have been spuriously introduced by the exponential decay model we used to estimate current PBB exposure or from small numbers. Nevertheless, we found that women who were 6–13 years old at the time of the Michigan incident (now 24–31 years) in the highest tertile and decile of current estimated PBBs had shorter bleed length relative to 36–38 year old women in the low tertile (n = 20). We restricted women in this study to those who were not transplacentally exposed to PBBs. Therefore, our sample was restricted to women who were exposed to PBBs only by the oral route. The age interaction we observed may indicate that women who were orally exposed before they reached menarche may have been affected during developmental maturation toward menses. Eskenazi et al reported longer cycle length in relation to higher levels of postnatal premenarcheal dioxin exposure, but no differences in bleed length [62]. Blanck et al found earlier age at menarche among a different sample of Michigan girls exposed both in utero to high maternal PBB levels and through breastfeeding [31]. These studies suggest that reproductive events may be affected by the timing and route of developmental and pre-pubertal exposure to halogenated hydrocarbons.

Laboratory assays of urine hormone levels have been useful to document concomitant ovulatory status with self-reported cycle length and bleed length [11,22]. Our reliance on self-reported menstrual cycle information alone is a limitation reported by others [10,63] and may be a source of misclassification. The resulting bias from selective over-reporting of certain days would most likely weight the resulting differences in cycle length toward the median (or toward the null hypothesis) (Figure 2). We did not observe digit preferences in the distribution of bleed length (Figure 3). Bleed length, normally shorter in duration compared to cycle length, may be less prone to recall bias. A larger sample and prospectively collected menstrual data would improve the reliability of these self-reported outcome measures.

We were also limited by our reliance on decay estimates of current PBB exposure. Uncertainties in the original decay model were previously described: lack of information on weight gain or loss during cohort follow-up; lack of fasting requirements for blood draws; lack of serum lipid standardization of the PBB measures; detection methods evolving over time; and most women having only two PBB measurements [30]. Strengths of the decay model over previous PBB half-life studies included: inclusion of PBB determinations over longer follow-up; inclusion of women at much lower initial PBB levels; inclusion of a much larger sample of women; and in addition to age and BMI, the inclusion of information on breastfeeding duration, smoking, and parity [30].

Eligibility requirements for our study differed from the decay model study. The decay analysis restricted inclusion to females who were at least 16 years of age. Our lower age bound only required women to have been born before the contamination episode. Our sensitivity analysis excluding females below 16 years found similar results to our full model. We also included women with enrollment PBB levels below 2 ppb (n = 112), essentially those women in our low enrollment tertile below the LOD (Table 2). It is possible that different kinetics apply at levels below the lower enrollment PBB inclusion bound of the decay model. The decay model found that half-life was shorter among women with low compared to high enrollment PBB levels [30]. Assuming the decay model holds for levels below 2 ppb, and assuming BMI constant, the current PBB estimates would not be biased. If the decay model fails below 2 ppb, third-order kinetics may apply. If the true half-life is shorter for enrollment PBBs below 2 ppb than above, then the decay model would overestimate current PBB levels, given a woman's BMI. In this case, women with low PBB levels at interview would be misclassified in higher adjusted PBB tertiles, and differences in cycle length or bleed length may be underestimated. If the true half-life is longer for enrollment PBBs below 2 ppb, then we would observe an overestimation of effect. We extrapolated our results further out to the time of the MFHS interview (22.4 years maximum) than the original decay model (11.1 years maximum). We acknowledge that this longer extrapolation is a limitation; however, data on long-term decay are not available. We believe that by using this decay model to estimate current PBB exposure that either no bias or an underestimation of effect is more likely, given the complex and nonlinear relationship between initial PBB level, BMI, and half-life described by Blanck et al [30].

Laboratory detection methods for PBBs changed over time to include PCB and pesticide determinations, and are described elsewhere [30,64], while early MDCH biologic sampling protocols did not require fasting blood draws nor standardization for serum lipids [2,60,64-68]. Nonfasting samples have higher mean concentrations of lipophilic compounds and higher total serum lipids than fasting samples. Postprandial increases in serum lipids have been shown to fluctuate with serum chlorinated hydrocarbon levels over a 24-hour period [69]. The lack of fasting requirements may add some measurement error in this present study; however; we believe that it would result in nondifferential misclassification. A woman's exposure status, even if known, is unlikely to prompt her decision to eat in relation to the time of a blood draw. Therefore, bias would most likely be toward the null hypothesis.

More recently, Schisterman et al proposed that lipid standardization is highly prone to bias, and advocate careful definitions of a causal framework for exposure, lipids, and health outcomes. There are study design frameworks when models not standardized for total lipids, equivalent to wet weight analyses, would be preferred [70]. Estrogens and other exogenous hormones are believed to alter plasma lipid and lipoprotein levels [71], and we hypothesize that potential endocrine disruptors, like PBBs or PCBs, may do the same. Since adiposity, energy homeostasis, and ovulation are believed to be intrinsically related [72], adjusting PBB or PCB levels for serum lipids could inappropriately adjust for the exposures themselves [70]. If this framework for the interrelationships holds true, then our lack of lipid standardization may not necessarily be a limitation. Statistical differences have been previously noted comparing lipid adjusted and wet weight analyses. When Cooper et al adjusted their menstrual cycle analyses for lipids, their serum-lipid analyses between PCBs with cycle length were somewhat attenuated compared to wet-weight analyses, but not with bleed length [10].

Our PBB exposure levels quantify only of the main congener, PBB 153. Different PBB congeners may have different endocrine-related effects related to menstrual function as suggested in some studies of PCBs [10,11] and by other proposed disruptors [62,73-78]. In congener-specific analyses, Cooper et al observed shorter cycle length with increasing exposure to PCB 52. This is in contrast to cycle lengthening when total PCBs are considered [10]. Windham et al noted that cycle length may decrease with increasing levels of PCB 187 but not with other congeners [11]. Rat and hamster models for follicular atresia and ovulatory delay with phenobarbital administration have been demonstrated [75-78], and PBB 153 is a phenobarbital-type inducer [27]. Among smokers, folliculotoxic effects of polycyclic aromatic hydrocarbons on primordial oocytes have been implicated in earlier menopause [79-82], while no relation between PBB exposure and time to menopause was found among women in the MFHS [83]. Other chemicals believed to alter endocrine function include dichlorodiphenyltrichloroethane (DDT), 1,1-dichloro-2,2-bis(*p*-chlorophenyl)ethylene (DDE), and polybrominated diphenyl ethers (PBDEs) [10,11,84,85]. The MFHS is limited by the lack of extensive information on these additional exposures.

## Conclusion

This study suggests that PBB exposure may impact ovarian function as indicated by menstrual cycle length and bleed length. However, these associations were found among the small number of women with recent weight loss suggesting either a chance finding or that mobilization of PBBs from lipid stores may be important. These results should be replicated with larger numbers of women exposed to lipophilic compounds.

## List of Abbreviations

BMI body mass index

CDF cumulative distribution function

DDE 1,1-dichloro-2,2-bis(*p*-chlorophenyl)ethylene

DDT dichlorodiphenyltrichloroethane

LOD limit of detection

MDCH Michigan Department of Community Health

MFHS Michigan Female Health Study

PBB polybrominated biphenyl

PBDE polybrominated diphenyl ether

PCB polychlorinated biphenyl

Ppb parts per billion

QQ quantile-quantile

## Competing interests

The author(s) declare that they have no competing interests.

## Authors' contributions

SID conceived, carried out, and interpreted the analytical models and drafted the manuscript. MM was Principal Investigator and PET, co-PI of the MFHS funded by the U.S. Environmental Protection Agency and the National Institute of Environmental Health Sciences. In that capacity they were responsible for scientific decisions regarding the study. LLC is PI of the Michigan PBB Long-Term Study at the MDCH. VSH oversaw the statistical analyses. MM, PET, HMB, CR and AKH, developed and implemented the protocol for the MFHS, assisted by LLC. HMB, MM, PET, AKH, and VSH conceived and developed the exponential decay mathematical model for the MFHS. LLC and the MDCH study staff provided the investigators access to the cohort, laboratory, and interview data archives on study participants and provided cohort outreach. All authors read and approved the final manuscript.
